# Coexistence of a Heterozygous Caveolin-3 Deletion and a Novel Dystrophin Gene Mutation in a Duchenne Muscular Dystrophy Patient

**DOI:** 10.7759/cureus.34704

**Published:** 2023-02-06

**Authors:** Muhammad W Khan, Syed Ali Raza, Madiha Raza, Eli Rogers, Rosario Maria S Riel-Romero

**Affiliations:** 1 Neurology, University of Rochester, Rochester, USA; 2 Neurology, Louisiana State University Health Sciences Center, Shreveport, USA; 3 Neurology, Ziauddin University, Karachi, PAK

**Keywords:** muscular dystrophy, pediatric, myopathy, dystrophin, duchenne, caveolin 3

## Abstract

Inherited muscular abnormalities are debilitating disorders that greatly diminish the quality of life in affected individuals. Mutations in proteins such as dystrophin and caveolin, which together with other proteins form structural connections between the cytoskeleton and the extracellular matrix, are frequently the culprit of muscular dystrophies. In this case report, we describe a patient with a novel pathogenic dystrophin mutation co-existing with a caveolin-3 deletion. While genetically composed of this unique combination, the patient phenotypically presented with a primary clinical manifestation of Duchenne muscular dystrophy (DMD) in contrast to other cases of dual mutations in dystrophin and dystrophin-associated proteins.

## Introduction

Inherited muscular dystrophies are a set of disorders caused by mutations in structural proteins which link intracellular actin to the extracellular matrix. Specifically, the mutations occur in either the actin-binding dystrophin protein or in the extracellular protein-binding dystrophin-associated protein complex (DAPC), which includes different structural proteins including caveolins [1-3].

Duchenne muscular dystrophy (DMD), an X-linked disorder affecting 3,600-6,000 live male births worldwide, results from a mutation in the dystrophin gene and is one of the most severe dystrophinopathies, leading to progressive muscle inflammation, weakness, and degeneration [1-4]. DMD is usually diagnosed by five years of age and mortality is typically reported in the second or third decade of life [1, 5, 6]. The severity of the disease manifestation is highly dependent on the type of mutation in the dystrophin gene.

The DMD gene codes for dystrophin which is a large, 427-kDa cytoskeletal protein whose primary function is linking actin to the DAPC. Specifically, this is accomplished by the amino-terminus of dystrophin binding to F-actin and the cysteine-rich region (located prior to the carboxyl-terminus) binding to the DAPC at the sarcolemma [7-10]. The DAPC is a large multi-component complex and is specifically comprised of dystroglycans, sarcoglycans, and caveolins, whose primary function is to tether the dystrophin-actin complex to the sarcolemma and extracellular matrix structure [11].

The caveolins are components of the DAPC that allow for the formation of caveolae, which are cave-like invaginations of the sarcolemma into the cytoplasm. Caveolin-3 (CAV3) is one such type of caveolin protein, and its associated caveolae contain many important macromolecules, including dystrophin-glycoprotein complex proteins, neuronal nitric oxide synthase (nNOS), phosphofructokinase, muscle-specific isoform (PFK-M), and dysferlin [12]. CAV3 is associated with a greater family of muscle disorders called caveolinopathies [13]. Here, we report on and discuss therapeutic approaches for an interesting case of the co-existence of a novel DMD mutation and a heterozygous CAV3 gene deletion in the same individual with primarily clinical manifestations of DMD.

## Case presentation

A nine-year-old, right-handed male child presented to our institute with a chief complaint of muscle weakness. The patient had a past medical history significant for hypospadias with repair, sickle cell trait, and prematurity, with delivery at 31 weeks via a caesarean section for maternal eclampsia. The pregnancy was additionally complicated by maternal sickle cell disease. The patient was the only child, and the mother had no history of miscarriages. The family neurologic history was significant for attention deficit hyperactive disorder in the mother; other family history was significant for sickle cell trait and sickle cell disease. Notably, no one in the family was known to have a neuromuscular disease or muscular dystrophy, although a complete pedigree was not possible to obtain due to a complex family social situation. The neonatal period was complicated by jaundice; bilirubin peaked at a total bilirubin of 1.6 mg/dL, and this was managed by hydration with no resultant sequelae. The patient met normal motor milestones through 2.5 years of age and had not the patient had no significant hospitalizations until age seven; however, after 2.5 years it was remarked that he always walked on his tiptoes and with in-toeing of the feet. This was also accompanied by fatigue and tachypnea with exertion. At age seven, the patient had falls and difficulty in walking endurance due to muscle weakness and was evaluated by a pediatric neurologist for the first time. Following an initial examination, the patient was noted to have shortened heel cords bilaterally and a walker was prescribed; tachypnea and occasional tachycardia related to exertion persisted. His age-appropriate gross motor skill milestones, specifically climbing stairs, hopping, and maintaining balance, were never reached.

At the age of seven years, the physical examination demonstrated the child to have a playful, attentive, and interactive personality. No cranial nerve abnormalities were identified. The musculoskeletal exam was notable for a positive Gower sign with bilateral calf hypertrophy, in addition to shortened feet and lumbar lordosis. No physiologic or pathologic abnormality was seen with deep tendon reflexes. Motor strength was antigravity (3/5) bilaterally in the lower extremities compared to a slightly better push against resistance (4/5) in the bilateral upper extremities. There was no significant difference between the proximal and distal muscle groups. There was a clear waddling gait and the patient was unable to run or climb up stairs.

**Figure 1:**
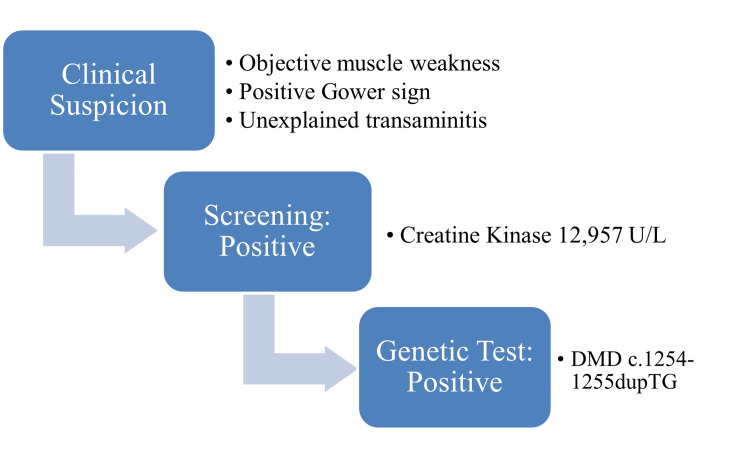
Diagnostic pathway graphic

A detailed examination revealed no signs of increased intracranial pressure and there was low suspicion of space-occupying lesions; thus, no imaging studies were performed. Pertinent lab tests included elevated creatine kinase at 12,957 U/L and mild transaminitis. The patient was diagnosed clinically with DMD based on this clinical history and laboratory and examination findings (Figure 1). Muscle biopsy and electromyography were not performed since they would be invasive, were not diagnostically necessary as per the American Association of Neurology guidelines on congenital muscular dystrophy [14] and were not thought to ultimately be able to alter the course of the disease or its management.

**Figure 2:**
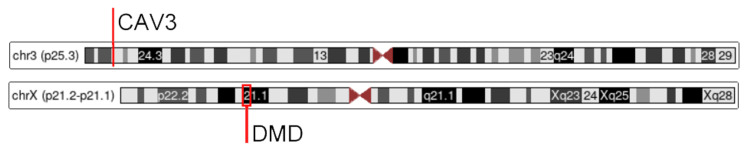
CAV3 and DMD genes CAV3 (Caveolin-3) and DMD (Duchenne muscular dystrophy) genes on the 3rd and X chromosomes, respectively. In this patient, the CAV3 site is heterozygously deleted and the DMD site contains a coding position 1254-1255 TG duplication causing a Glutamate to Valine shift at codon 419 with a concurrent frameshift. Images adapted from the UCSC Genome Browser.

Genetic testing using a comprehensive neuromuscular disorders panel (Invitae, San Francisco, USA) with next-generation sequencing demonstrated a pathogenic de novo variant in the DMD gene in Exon 11, characterized as c.1254-1255dupTG (p.Glu419Valfs*7). The patient was also found to have a pathogenic single copy deletion of CAV3 at site p25.3 (Figure 2). Further investigation revealed the presence of an identical CAV3 deletion in the mother who was asymptomatic by history, physical exam, and basic laboratory testing, although she did have a mildly elevated CK of 141 U/L. An echocardiogram of the patient revealed no wall motion or gross abnormalities. The patient’s clinical course was complicated only by dysphagia and a weak cough treated by outpatient PPI and physiotherapy.

## Discussion

Loss of function variants in the CAV3 gene has been previously reported as pathogenic [15,16]. A CAV3 deletion has never been reported previously in concert with a DMD gene mutation. Only one prior report exists demonstrating the coexistence of a missense CAV3 mutation specifically of NM_033337.2:c.80G>A and total in-frame deletion of DMD exons 45-55 in a proband with Becker muscular dystrophy (BMD) and rippling disease [17]. However, the patient in that study had a different phenotype including percussion-induced muscle mounding and generalized muscle stiffness with a negative Gower sign, likely due to the intact reading frame for the DMD gene despite the large deletion. The patient described in our study appeared to have a more classic phenotypic presentation of DMD, likely due to the frameshifting resulting from the duplication, with no sign of Rippling Disease. While initially reassuring that this heterozygous CAV3 deletion may manifest recessively given the patient’s grossly asymptomatic mother, her mildly elevated CK is concerning for some manifestation of the heterozygous deletion, and a potentially late-presenting additive effect from the interaction of these dual CAV3 and DMD mutations is possible and would be concerning for accelerated clinical decline. It is critical to closely monitor this patient’s course, especially by echocardiograms and EKGs considering the association of cardiomyopathy and QT-prolongation with CAV3 mutations [13].

There are many therapeutic approaches for DMD specifically, but these are not used for CAV3-pertinent mutations and the efficacy on an individual with dual mutations is unknown. A major recent gene therapy approach is known as exon-skipping. This approach relies on the fact that DMD patients could essentially produce BMD-like dystrophin, whereby target exons are skipped while maintaining the reading frame and critical binding domains, thus enabling the production of partially functioning dystrophin rather than the production of non-functional dystrophin. Antisense oligonucleotides (ASOs) have been employed to achieve this, whereby the ASOs bind to the target exon in the pre-mRNA transcript, altering mRNA splicing and thus leading to skipping of target exon. Another approach with possible applicability in the future involves adeno-associated virus (AAV) delivery systems, whereby microdystrophins may be delivered as gene therapy to patients [18]. If the micro- or mini-dystrophins retain critical functional binding sites, they could be a potential therapeutic approach for our patient as well. Preliminary data employing dual-AAV gene therapy in canine-DMD models showed favorable results [19]; however, this therapy currently requires systemic high-dose AAV administration, and subsequent translation into humans is less practical at this time [18]. Hypothetically, an interesting treatment choice in this patient would be the application of the “stop codon readthrough” approach such as with ataluren, which decreases ribosomal sensitivity to premature stop codons and thereby enables translation to continue. Given this patient’s frameshift at exon 11, it is possible that a stop codon readthrough could produce an altered remainder of the protein which may grant partial functionality, although it is also possible that the frameshifted remainder of the protein would be non-functional or even pathologic and there are no data as of yet to support this supposition. This remains a potential future treatment approach if it were to confer some restoration of function for the DMD protein.

## Conclusions

In this case report, we present a patient with DMD who has a novel deleterious DMD exon 11 frameshift mutation variant and a concomitant heterozygous CAV3 single copy deletion. To our knowledge, there has been one prior report demonstrating the coexistence of missense CAV3 mutation and total exon 45-55 DMD deletions, which had a clinical presentation primarily of rippling disease rather than DMD. With advancements in our genetic toolkit, we can expect patient-specific treatment modalities to emerge which would address specific mutations and lead to the alleviation of disorders. Our patient’s primary clinical manifestation was of DMD which allows us to consider DMD-targeted therapies; however, it is critical to remain cognizant that these therapies will not target the co-existing CAV3 deletion if it proves to interact with the DMD gene, and monitoring for long-term outcomes associated with CAV3 deletion remains paramount. With advancements in our genetic toolkit, we can expect patient-specific treatment modalities to emerge which would address specific mutations and lead to the alleviation of such disorders.
